# Autonomous Artificial Intelligence Increases Access and Health Equity in Underserved Populations with Diabetes

**DOI:** 10.21203/rs.3.rs-3979992/v1

**Published:** 2024-03-13

**Authors:** T.Y. Alvin Liu, Jane Huang, Roomasa Channa, Risa Wolf, Yiwen Dong, Mavis Liang, Jiangxia Wang, Michael Abramoff

**Affiliations:** Johns Hopkins University; Johns Hopkins School of Medicine; University of Wisconsin; Johns Hopkins University School of Medicine; Johns Hopkins University; Johns Hopkins University; Johns Hopkins University; University of Iowa

**Keywords:** Autonomous artificial intelligence, diabetic eye disease, diabetic retinopathy, health equity, health access, adherence

## Abstract

Diabetic eye disease (DED) is a leading cause of blindness in the world. Early detection and treatment of DED have been shown to be both sight-saving and cost-effective. As such, annual testing for DED is recommended for adults with diabetes and is a Healthcare Effectiveness Data and Information Set (HEDIS) measure. However, adherence to this guideline has historically been low, and access to this sight-saving intervention has particularly been limited for specific populations, such as Black or African American patients. In 2018, the US Food and Drug Agency (FDA) De Novo cleared autonomous artificial intelligence (AI) for diagnosing DED in a primary care setting. In 2020, Johns Hopkins Medicine (JHM), an integrated healthcare system with over 30 primary care sites, began deploying autonomous AI for DED testing in some of its primary care clinics. In this retrospective study, we aimed to determine whether autonomous AI implementation was associated with increased adherence to annual DED testing, and whether this was different for specific populations. JHM primary care sites were categorized as “non-AI” sites (sites with no autonomous AI deployment over the study period and where patients are referred to eyecare for DED testing) or “AI-switched” sites (sites that did not have autonomous AI testing in 2019 but did by 2021). We conducted a difference-in-difference analysis using a logistic regression model to compare change in adherence rates from 2019 to 2021 between non-AI and AI-switched sites. Our study included all adult patients with diabetes managed within our health system (17,674 patients for the 2019 cohort and 17,590 patients for the 2021 cohort) and has three major findings. First, after controlling for a wide range of potential confounders, our regression analysis demonstrated that the odds ratio of adherence at AI-switched sites was 36% higher than that of non-AI sites, suggesting that there was a higher increase in DED testing between 2019 and 2021 at AI-switched sites than at non-AI sites. Second, our data suggested autonomous AI improved access for historically disadvantaged populations. The adherence rate for Black/African Americans increased by 11.9% within AI-switched sites whereas it decreased by 1.2% within non-AI sites over the same time frame. Third, the data suggest that autonomous AI improved health equity by closing care gaps. For example, in 2019, a large adherence rate gap existed between Asian Americans and Black/African Americans (61.1% vs. 45.5%). This 15.6% gap shrank to 3.5% by 2021. In summary, our real-world deployment results in a large integrated healthcare system suggest that autonomous AI improves adherence to a HEDIS measure, patient access, and health equity for patients with diabetes – particularly in historically disadvantaged patient groups. While our findings are encouraging, they will need to be replicated and validated in a prospective manner across more diverse settings.

## Introduction

Diabetic eye disease (DED) affects a third of people with diabetes mellitus (DM) and is a leading cause of blindness and visual impairment in working-aged adults in the developed world.^1^ Since patients with DED often have no symptoms in the early stages of disease, current guidelines from the American Academy of Ophthalmology and American Diabetes Association recommend that patients with diabetes receive an annual eye examination.^2,3^ These annual screenings allow for early diagnosis and treatment that can help prevent severe vision loss.^4^ As such, annual DED testing is included as a Healthcare Effectiveness Data and Information Set (HEDIS) measure.

Unfortunately, adherence to these annual DED testing guidelines has historically been low. A previous study in the United States found that only 50–60% of Medicare beneficiaries with DM received annual eye exams.^5^ This adherence rate is even lower (30–40%) in smaller health systems and low-income metropolitan patient populations.^6,7^ Previous qualitative investigations have identified misinformation about the importance of regular testing, logistic challenges with scheduling appointments, and anxiety as key barriers to annual eye exams.^8^ In 2018, the FDA De Novo authorized the use of an autonomous artificial intelligence (AI) system (LumineticsCore^®^, Digital Diagnostics, Coralville, IA) to diagnose DED. This system can autonomously analyze images of the retina at the point of care, such as at primary care clinics, with a sensitivity of 87.2% and specificity of 90.7% in a pivotal clinical trial against a prognostic standard, i.e. patient outcome.^9^ Subsequent studies have further validated the accuracy, sensitivity, and specificity of this autonomous AI technology, using ophthalmologists’ reads as the reference standard.^10–12^

In 2020, Johns Hopkins Medicine, an integrated healthcare system with over 30 primary care sites, began deploying autonomous AI for DED testing in some of its primary care clinics. This study aimed to determine whether implementation of this technology increased the rate of care gap closure in different patient populations and whether its implementation was associated with higher overall adherence rates.

## Results

### Patient demographics

A total of 17,674 patients with diabetes were managed at Johns Hopkins Medicine in 2019. Most patients were female (53.0%) and under 65 years old (69.0%). The two most highly represented racial groups were White (45.2%) and Black or African American (40.6%), and the two most common insurance coverages were commercial/other (48.7%) and Medicare (30.0%). Nearly all patients resided in an urban setting.

A total of 17,590 patients with diabetes were managed at Johns Hopkins Medicine in 2021. Again, most patients were female (51.1%) and under 65 years old (71.1%). Most patients were White (47.9%) and Black or African American (37.1%), and most patients had either commercial/other insurance (53.1%) or Medicare (28.1%). A detailed breakdown of the population demographics is available in Table 1. Overall, the patient demographics between AI-switched sites and non-AI sites were similar. Patients at non-AI sites had a higher inflation-adjusted mean income ($90,200 in 2019 and $98,000 in 2021) than patients at AI-switched sites ($63,400 in 2019 and $63,400 in 2021). Additionally, more patients at non-AI sites were covered under military insurance compared to AI sites (13.0% vs. 6.2%). These differences were fixed as covariates in subsequent statistical analysis to avoid confounding.

### Changes in DED adherence rate from 2019 to 2021

In 2019, the overall adherence rate across all sites was 42.2%. The baseline adherence rate was 46.1% at AI-switched sites and 40.4% at non-AI sites. In 2021, the overall adherence rate across all sites increased to 44.8%. Of note, the adherence rate increased to 54.5% at AI-switched sites and remained stable at 40.3% at non-AI sites. A detailed breakdown in adherence rate for each patient population is shown in Table 2.

We examined the change in DED adherence rate across 8 demographic and social determinants of health categories: gender, age, race, ethnicity, language preference, insurance coverage, geography, and national ADI quartile (Table 2). The largest adherence gaps at AI-switched sites in 2019 had significantly decreased by 2021 after AI implementation. For the race category, the largest initial gap in 2019 was between American Indian/Alaska Native patients and Native Hawaiian/Other Pacific Islander patients. This gap shrank from 37.3% in 2019 to 20.5% in 2021. For the insurance category, the largest initial gap in 2019 was between patients with military insurance and those covered by Medicaid. This gap shrank from 32.9% in 2019 to 21.1% in 2021. For the ADI category, the largest initial gap in 2019 was between the 1st and 2nd ADI quartiles. This gap shrank from 8.8% in 2019 to −2.5% in 2021. From 2019 to 2021, among the AI-switched sites, the largest improvements in adherence rate within the categories of race, insurance coverage and ADI quartile were: +19.5% in Native Hawaiian/Other Pacific Islander patients, + 11.9% in Black/African American, + 13.6% in Medicaid-insured patients, and + 11.3% in the 2nd ADI quartile.

### Logistic regression model analyses

A total of 12,660 patients that were evaluated in primary care clinics in both 2019 and 2021 were included in the following analyses. Our model controlled for possible confounding factors, including social determinants of health. The odds ratio of DED testing adherence in 2021 compared to 2019 among AI-switched sites was 1.54 (95% CI: [1.42, 1.66], p < 0.001). Among non-AI sites, this odds ratio was 1.13 (95% CI: [1.07, 1.19], p < 0.001). The calculated odds ratio between AI-switched sites and non-AI sites was 1.36 (95% CI: [1.24, 1.50], p < 0.001). In other words, the odds ratio of adherence at AI-switched sites was 36% higher than that of non-AI sites which still relied on traditional screening methods, suggesting that there was a higher increase in DED testing between 2019 and 2021 at AI-switched sites than at non-AI sites.

There were 7,365 patients total who were non-adherent in 2019 but became adherent in 2021. Of these patients, the odds ratio of being managed at an AI-switched vs. non-AI site was 2.07 (95% CI: [1.85, 2.32], p < 0.001).

## Discussion

In this study, we examined the change in annual DED testing adherence rate before and after implementation of autonomous AI technology at primary care clinics at Johns Hopkins Medicine. From 2019 to 2021, i.e. coming out of the COVID pandemic which caused widespread adherence issues, we observed a substantial increase in adherence rate among AI-switched sites, while the adherence rate among non-AI sites remained unchanged. This improvement in AI-switched sites over non-AI sites remained statistically significant after adjusting for potentially confounding social determinants of health in our logistics regression model. This observation was further bolstered by the fact that “convertors,” patients who were not adherent in 2019 but became adherent in 2021, were two times more likely to be managed at an AI-switched site than a non-AI site. Among the AI-switched sites, the patient populations that experienced substantial improvement in adherence rate included Black or African American patients, patients with Medicaid insurance coverage, and patients with high ADI scores. Therefore, our data suggests that deployment of autonomous AI improved access and health equity in these historically disadvantaged patient groups. Our additional observations are as follows.

First, the overall adherence rate across all sites was 42.2% in 2019, which was lower than the nationwide average of 58.3%,^13^ but higher than the 34% seen in other low-income metropolitan populations in the United States.^3^ The overall adherence rate in 2021 increased slightly to 44.8%. However, the adherence rate among AI-switched sites substantially increased to 54.5%, much closer to the nationwide average. By extrapolation of our data, large scale deployment of this technology across the entire health system could substantially increase overall adherence rate, which in turn could improve HEDIS metrics, Centers for Medicare and Medicaid Services (CMS) Merit-based Incentive Payment System (MIPS) rating, and payer reimbursement.

Second, among the AI-switched sites, there were significant outsize increases in adherence rates in the Black or African American (+ 12%) and Native Hawaiian or Other Pacific Islander (+ 20%) patient populations from 2019 to 2021. In contrast, over the same time period, the adherence rates for these two patient groups actually decreased by 1% and 2%, respectively, among non-AI sites. These data suggest that the deployment of autonomous AI improved access when it comes to DED management, particularly for historically disadvantaged populations. Prior studies have found that the African American population uses eye care services at much lower rates than White patients, even though Black patients with type 2 diabetes are significantly more likely to develop retinopathy than White patients.^14–17^ A previous focus group found that for many African American patients, transportation, lack of free time, and inconvenience of eye exam served as main barriers for annual DED screening.^17^ In fact, minority populations overall have consistently lower unadjusted eye examination rates than White populations.^18^

Third, our data also suggest that autonomous AI improved health equity by closing care gaps between patient groups. Among AI-switched sites and before AI deployment in 2019, a large adherence rate gap existed between Asian Americans and Black/African Americans (61.1% vs. 45.5%). This 15.6% gap shrunk to 3.5% by 2021. Similarly, among AI-switched sites and before AI deployment in 2019, a large adherence gap existed between patients with military insurance and patients with Medicaid insurance (63.1% vs. 30.2%). This 32.9% gap shrunk to 21.1% by 2021. Lastly, among AI-switched sites and before AI deployment in 2019, an adherence rate gap of 8.4% existed between the most socioeconomically advantaged (ADI 1st quartile) and the most socioeconomically disadvantaged (ADI 4th quartile). By 2021, the adherence rate gap between patients from all 4 quartiles had closed.

Fourth, we observed a ceiling effect with improvement in adherence rate even after autonomous AI deployment. Though autonomous AI improved access for the most disadvantaged patient groups and reduced care gaps, such improvement was not universal. For example, among the AI-switched sites, multiple patient subgroups barely experienced any improvement in adherence rate from 2019 to 2021: Asian (−0.2%), American Indian or Alaska Native (+ 2.7%), non-English speaking patients (+ 2.2%), and military insurance coverage (+ 1.8%). While it is beyond the scope of the current study to evaluate the cause for this ceiling effect, the relative lack of trust in healthcare AI or concerns about data usage by patients could be a limiting factor in adoption.^19–21^ Patient trust in autonomous AI technologies will affect adoption of AI into our healthcare system, and this is certainly an important topic that warrants more thorough investigation.

Our study is limited by its retrospective nature and the fact that nearly all patients included in our study live in a metropolitan area, so our observations may not generalize to patient populations living in micropolitan, small town, and rural residences. However, our data demonstrated that deployment of autonomous AI for DED testing in the primary care setting is highly associated with improvement in adherence rate, patient access and health equity. Future studies in the form of a prospective randomized clinical trial, similar to a recent study that investigated the role of autonomous AI for DED testing in youth with diabetes^22^, could help delineate whether there is a causal relationship between autonomous AI and improvement in population-level metrics. Additionally, qualitative surveys that evaluate patient views toward the use of AI technology could help identify targets of patient education that can help address the ceiling effect observed in our study.

## Methods

This was a retrospective study approved by the Institutional Review Board of the Johns Hopkins School of Medicine. All research adhered to the tenets of the Declaration of Helsinki. Our study included all patients with diabetes mellitus who were managed at primary care sites of Johns Hopkins Medicine in the calendar years 2019 (pre-AI deployment) and 2021 (post-AI deployment). Subject demographic information that was retrieved from the electronic health records system included gender, age, race, ethnicity, preferred language, insurance status, ZIP code of residence, national area deprivation index (ADI), and inflation-adjusted median household income in the past 12 months.

Additionally, from the ZIP code data, each patient’s residence was identified as metropolitan, micropolitan, small town, or rural based on the 2010 Rural-Urban Commuting Area Codes.^23^ The national ADI score is a 100-point scale that measures socioeconomic disadvantage based on data like a geographic region’s housing quality, education, income, and employment.^24^ A higher national ADI score correlates with increased overall socioeconomic disadvantage.

Each patient’s clinic site was categorized as either “AI-switched” or “non-AI” site. An “AI-switched” site is defined as a site that did not have autonomous AI DED testing (LumineticsCore^®^, Digital Diagnostics, Coralville, IA) in 2019, but had it by 2021. A “non-AI” site is defined as a site that never had autonomous AI deployment and where patients are referred to eyecare for DED testing.

### Statistical Analyses

Patient demographic characteristics were described. Categorical and binary variables were reported as numbers and percentages. These variables were compared using chi-squared tests.

The primary outcome measure was adherence to DED testing in a given calendar year. Adherence was defined as either receiving an autonomous AI exam or a dilated eye exam with an ophthalmologist. The odds ratio of adherence to DED testing in 2021 compared to 2019 at AI-switched sites was calculated. The odds ratio of adherence to DED testing in 2021 compared to 2019 at non-AI sites was calculated. Then, a difference-in-difference analysis was conducted to compare DED testing adherence across patient groups in 2019 and 2021. A logistic regression model was fitted using an exchangeable correlation structure with a constant variance between 2019 and 2021. A robust variance was estimated using the Generalized Estimating Equations framework. In this model, predictors included clinic site (AI-switched vs. non-AI), year of visit (2019 vs. 2021), and the interaction between clinic site and year of visit. Fixed covariates included the following patient characteristics: race, ethnicity, gender, insurance type, census bureau geographic area, national ADI, and inflation-adjusted median household income in the past 12 months. All patients included in this difference-in-difference analysis were paired across the two years, meaning they each had a primary care visit in both 2019 and 2021.

A subset analysis was performed for patients who were non-adherent in 2019 but were adherent in 2021. To investigate whether being managed at an AI-switched site is associated with transitioning from being non-adherent to adherent, a logistic regression model was fit with the adherence outcome in 2021 as a function of clinic site (AI-switched vs. non-AI), while controlling for the same covariates as above.

All statistical analysis was performed using R Statistical Software Version 4.3.1 (R Foundation for Statistical Computing, Vienna, Austria). The level of statistical significance was set at p<0.05.

## Figures and Tables

**Table 1:** Patient demographics of AI-switched sites and non-AI sites in 2019 and 2021

	2019		2021	
N (%)	AI-switched sites	Non-AI sites	AI-switched sites	Non-AI sites
**Total patients**	5505	12169	5580	12010
**Gender**				
Female	3037 (55.2)	6334 (52.1)	3040 (54.5)	5989 (49.9)
Male	2468 (44.8)	5835 (47.9)	2540 (45.5)	6021 (50.1)
**Age**				
Under 65 years	3730 (67.8)	8470 (69.6)	3982 (71.4)	8580 (71.4)
65 years and over	1775 (32.2)	3699 (30.4)	1598 (28.6)	3430 (28.6)
**Race**				
Black or African American	2606 (47.3)	4563 (37.5)	2648 (47.5)	3907 (32.5)
White	2411 (43.8)	5581 (45.9)	2402 (43.0)	5964 (49.7)
Other	258 (4.7)	1150 (9.5)	288 (5.2)	1121 (9.3)
Asian	190 (3.5)	783 (6.4)	202 (3.6)	922 (7.7)
American Indian or Alaska Native	25 (0.4)	64 (0.5)	27 (0.5)	67 (0.6)
Native Hawaiian or Other Pacific Islander	15 (0.3)	28 (0.2)	13 (0.2)	29 (0.2)
**Ethnicity**				
Not Hispanic or Latino	5253 (95.4)	11108 (91.3)	5297 (94.9)	11024 (91.8)
Hispanic or Latino	139 (2.5)	672 (5.5)	159 (2.9)	594 (5.0)
Unknown	113 (2.1)	389 (3.2)	124 (2.2)	392 (3.2)
**Language Preference**				
English	5393 (98.0)	11679 (96.0)	5450 (97.7)	11687 (97.3)
Non-English	112 (2.0)	490 (4.0)	130 (2.3)	323 (2.7)
**Insurance Coverage**				
Commercial and Other	2823 (51.3)	5780 (47.5)	2977 (53.4)	6408 (53.4)
Pure Medicare	1738 (31.6)	3554 (29.2)	1630 (29.2)	3336 (27.8)
Military	339 (6.1)	1584 (13.0)	325 (5.8)	1585 (13.2)
Medicaid	295 (5.4)	646 (5.3)	329 (5.9)	332 (2.8)
Medicare Advantage	246 (4.5)	285 (2.4)	243 (4.3)	244 (2.0)
Self-Pay	64 (1.1)	320 (2.6)	76 (1.4)	105 (0.8)
**Geography**				
Metropolitan	5494 (99.8)	12147 (99.8)	5569 (99.8)	11990 (99.8)
Micropolitan	10 (0.2)	11 (0.1)	10 (0.2)	13 (0.1)
Small Town	1 (0.0)	9 (0.1)	1 (0)	6 (0.1)
Rural	0	2 (0)	0	1 (0)
**National ADI**				
1st Quartile (1–25)	417 (7.6)	3767 (31.0)	430 (7.7)	4298 (35.8)
2nd Quartile (26–50)	943 (17.1)	3453 (28.3)	958 (17.2)	3812 (31.7)
3rd Quartile (51–75)	1973 (35.8)	2553 (21.0)	2015 (36.1)	2524 (21.0)
4th Quartile (76–100)	2172 (39.5)	2396 (19.7)	2177 (39.0)	1376 (11.5)

**Table 2:** Change in annual DED testing adherence rate from 2019 to 2021 by demographic subgroups

	2019		2021		Change from 2019 to 2021	
	AI-switched sites	Non-AI sites	AI-switched sites	Non-AI sites	AI-switched sites	Non-AI sites
**Overall Adherence Rate**	46.1%	40.4%	54.5%	40.3%	+ 8.4%	−0.1 %
**Gender**						
Male	44.6%	39.9%	54.8%	39.6%	+ 10.2%	−0.3%
Female	47.3%	41.0%	54.3%	40.9%	+ 7.0%	−0.1 %
**Age**						
65 years and over	57.1%	52.6%	64.6%	49.9%	+ 7.5%	−2.7%
Under 65 years	40.9%	35.1%	50.5%	36.4%	+ 9.6%	+ 1.3%
**Race**						
American Indian or Alaska Native	64.0%	39.1%	66.7%	43.3%	+ 2.7%	+ 4.2%
Asian	61.1%	43.4%	60.9%	41.3%	−0.2%	−2.1 %
Black or African American	45.5%	40.9%	57.4%	39.7%	+ 11.9%	−1.2%
Native Hawaiian or Other Pacific Islander	26.7%	50.0%	46.20%	48.3%	+ 19.5%	−1.7%
White	45.7%	40.5%	51.0%	40.4%	+ 5.3%	−0.1 %
Other	45.0%	36.3%	52.4%	40.5%	+ 7.4%	+ 4.2%
**Ethnicity**						
Hispanic or Latino	46.8%	35.7%	49.1%	38.6%	+ 2.3%	+ 2.9%
Not Hispanic or Latino	46.3%	40.7%	54.8%	40.6%	+ 8.5%	−0.1 %
Unknown	38.1%	42.4%	51.6%	34.2%	+ 13.5%	−8.2%
**Language Preference**						
English	46.1%	40.6%	54.7%	40.3%	+ 8.6%	−0.3%
Non-English	45.5%	37.1%	47.7%	37.5%	+ 2.2%	+ 0.4%
**Insurance Coverage**						
Pure Medicare	49.9%	43.9%	58.0%	42.3%	+ 8.1%	−1.6%
Medicare Advantage	50.4%	53.7%	60.5%	46.3%	+ 10.1%	−7.4%
Medicaid	30.2%	36.2%	43.8%	31.6%	+ 13.6%	−4.6%
Military	63.1%	50.8%	64.9%	50.0%	+ 1.8%	−0.8%
Self-Pay	42.2%	37.5%	44.7%	37.1%	+ 2.5%	−0.4%
Commercial and Other	43.1%	35.5%	52.5%	37.0%	+ 9.4%	+ 1.5%
**Geography**						
Metropolitan	46.1%	40.5%	54.5%	40.3%	+ 8.4%	−0.2%
Micropolitan	30.0%	27.3%	91.0%	23.1%	+ 61.0%	−4.2%
Small Town	0%	33.3%	0%	50.0%	0%	+ 16.7%
Rural	NA	0%	NA	0%	NA	0%
**National ADI**						
1st Quartile (1 –25)	53.7%	39.5%	53.7%	40.0%	0%	+ 0.5%
2nd Quartile (26–50)	44.9%	40.3%	56.2%	40.8%	+ 11.3%	+ 0.5%
3rd Quartile (51–75)	46.0%	41.0%	53.5%	40.9%	+ 7.5%	−0.1 %
4th Quartile (76–100)	45.3%	41.5%	54.9%	38.4%	+ 9.6%	−3.1 %

## Data Availability

The original data will be available upon official written request to the corresponding author.
